# An Atypical Etiology of Suprasphincteric Fistula: A Forgotten Surgical Material

**DOI:** 10.1155/2010/189846

**Published:** 2010-06-17

**Authors:** Melih Paksoy, Volkan Ozben, Fadil Ayan, Arife Simsek

**Affiliations:** Department of General Surgery, Cerrahpasa Medical School, Istanbul University, Cerrahpasa, Fatih 34098, Istanbul, Turkey

## Abstract

While the majority of fistulas in ano result from infection of the anal crypts, complex, recurrent, and/or nonhealing fistulas should always raise the suspicion of a chronic underlying condition. In this paper, we present a 30-year-old male patient with a diagnosis of a complex suprasphincteric fistula caused by a surgical thread left behind after an orthopedic hip operation performed sixteen years ago. Partial fistulectomy, extraction of the foreign material, and debridement procedures were performed. Few cases of such complex fistulas in ano due to foreign materials have been described in the literature. After careful history-taking, meticulous physical examination under general anesthesia should be done in order to deal with this rare type of fistula.

## 1. Introduction

Fistula in ano is a common malady for patients and sometimes a very difficult problem for surgeons to deal with. It occurs secondarily to infection of the cryptoglands in most cases. However, if the condition is chronic, complex, or recurrent, an underlying pathology should be searched first in order to make correct decisions in the diagnosis and treatment. Cases of complex fistula in ano due to foreign bodies, although very rare, have been described in [1–3]. Foreign bodies may remain unrecognized and present themselves years later with complications. In this paper, we present a case of a suprasphincteric fistula resulting from a foreign body discovered in a patient who underwent a left hip joint operation 16 years ago.

## 2. Case Report

**Figure 1:**
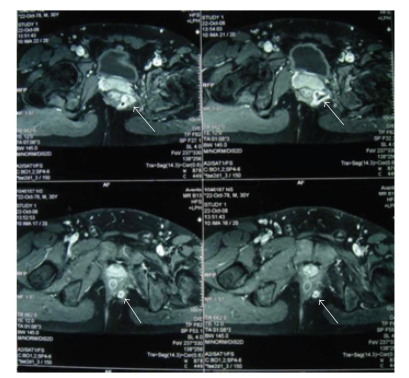
The pelvic MRI of the patient demonstrating the suprasphincteric fistula in ano with abscess formation (arrows).

**Figure 2:**
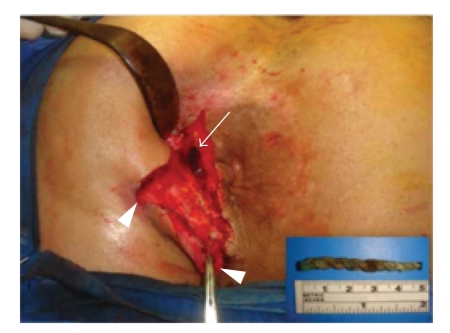
Intraoperative view of the external openings of the fistula in ano (arrow-heads) and the upper part of the tract where the foreign material resided (arrow). The extracted nonabsorbable braided thread is shown in the inlet figure.

**Figure 3:**
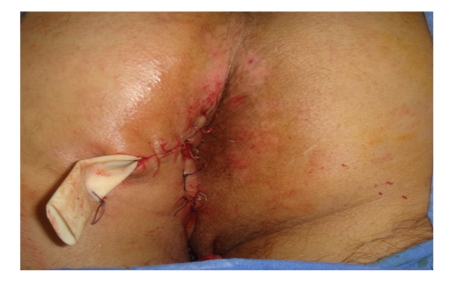
Postoperative view.

A 30-year-old male patient was electively admitted to our general surgery clinic in June 2009 with a diagnosis of fistula in ano. His past medical history revealed a serious fall during a football game which resulted in a left acetabular fracture in 1993. Open reposition and internal fixation procedures had been performed two months after this accident. Postoperatively, he had developed abscesses on the upper medial aspect of his left thigh in the second month and on the lateral aspect of the left gluteal region in the third month. All these abscesses had been treated by surgical drainage and with antibiotics. Three years after the hip operation, he had developed a perianal abscess which was also drained. However, the intermittent spontaneous purulent discharge in the perianal region continued for 13 years since the patient did not seek any medical care during this period. His past medical history was not conclusive for any inflammatory bowel disease. Pelvic magnetic resonance imaging (MRI) performed in a different medical center eight months prior to his admission demonstrated an abscess cavity, measuring 2 cm in diameter, located superior to the levator ani muscle and its tract of 10 cm coursing from its internal opening in the rectum down to the perianal region (Figure 1). The laboratory analysis including complete blood count, blood biochemistry, and erythrocyte sedimentation rate were within normal limits. Physical examination revealed the presence of two external openings 7 cm and 3 cm from the anal verge at the 3 o'clock and 2 o'clock positions, respectively. The results of digital rectal examination and colonoscopy were normal and the internal opening of the tract was not visualized. Based on these clinical findings, surgical intervention for the complex fistula in ano was planned. After bowel preparation, the patient was taken to the operation room. Under general anesthesia, the patient was placed in a jack-knife position and the buttocks were taped apart. The fistula tracts were laid open to the level of the anal sphincters. At this level, digital examination was performed towards the upper part of the tract and a foreign body located above the levator ani muscle was felt. A nonabsorbable braided thread of 5 cm in length and 6 mm in thickness was extracted (Figure 2). After partial excision and curretage of the tract, a penrose drain was placed and the wound was closed with interrupted simple absorbable sutures (Figure 3). Following the discovery of this object, a plain pelvic radiography was performed, this demonstrated advanced left acetabular degeneration with no other foreign body visible. Following an uneventful postoperative recovery, the patient was discharged home on the third postoperative day. On follow-up visits, complete resolution of the perianal fistula was observed after a month. The patient remained symptom-free during the 7-month follow-up period.

## 3. Discussion

Fistula in ano is a granulating tract between the anorectum and the perianal region or perineum. A typical fistula may consist of a tract, a primary (internal) opening and a secondary (external) opening. It is characterized by frequent discharge through the external opening. Sometimes the tract becomes occluded and a sinus remains. Therefore, the perianal sinus should be considered as another form of perianal fistula [4]. In the presented case, the pelvic MRI of the patient delineated the suprasphincteric type of fistula with no evidence of a foreign material eight months prior to his admission. It seems likely that the internal opening in the rectum was obliterated over time due to the foreign body reaction and the drainage of the abscess continued through the external openings.

While 90% of fistulas in ano are cryptoglandular in origin, specific infections such as tuberculosis, actinomycosis, lymphogranuloma venereum, Crohn's disease, ulcerative rectocolitis, trauma, foreign bodies, malignant tumors of the rectum, prostate, bladder, uterus or anus, Hodgkin's disease, leukemia, and postradiotherapy have also been recognized as etiological factors [5].

In the literature, only a few such fistula in ano cases due to foreign bodies have been reported. Hasan et al. [2] reported an unusual case which concerns a Filshie clip which is used to ligate the separated ends of the tubes for the sterilization of women. Migration of this clip was reported to cause a perianal abscess and later a fistula 12 years after its application. In a case similar to this, Dua and Dworkin [3] presented another patient with a fistula in ano and an ischiorectal abscess due to a Filshie clip 3 years after laparoscopic tube ligation. Byrne et al. [6] presented two interesting cases in which they described rectal perforation and ischiorectal abscesses caused by a foreign body (ingested bones) in the rectum. Lastly, Cash et al. [7] also described a perianal fistula due to a chicken bone. 

It seems likely that the forgotten surgical thread during the hip operation of the patient first caused the recurrent attacks of abscesses in the gluteal and thigh regions and later resulted in the ischiorectal abscess and suprasphincteric fistula over the years. To the best of our knowledge, this is the first case of fistula in ano caused by a forgotten surgical material after an orthopedic operation. 

In the presented case, neither colonoscopy nor pelvic radiography or MRI could reveal the forgotten surgical thread as an etiology of the fistula in ano. For such cases, endorectal ultrasonography should also be considered as a complementary diagnostic tool in order to detect any associated foreign material in patients with complex and nonhealing fistula in ano.

In order to maintain anal continence, we performed partial fistulectomy to the level of the anal sphincters and debrided the fistulous tract above the level of sphincters. During the postoperative follow-up period, complete resolution of the fistula, without any compromise of sphincter function or any further discharge, have been observed.

Complex, recurrent, or nonhealing perianal fistula should always raise the suspicion of a chronic underlying condition. Foreign bodies, although very rare, should also be borne in mind especially in patients with a history of pelvic operations. Past medical history and physical examination of the patients with complex fistula in ano should be carefully performed so as not to misdiagnose this rare etiology.
